# The ^68^Ga/^177^Lu-theragnostic concept in PSMA-targeting of metastatic castration–resistant prostate cancer: impact of post-therapeutic whole-body scintigraphy in the follow-up

**DOI:** 10.1007/s00259-019-04583-2

**Published:** 2019-11-27

**Authors:** Johanna Maffey-Steffan, Lorenza Scarpa, Anna Svirydenka, Bernhard Nilica, Christian Mair, Sabine Buxbaum, Jasmin Bektic, Elisabeth von Guggenberg, Christian Uprimny, Wolfgang Horninger, Irene Virgolini

**Affiliations:** 1 Department of Nuclear Medicine, Medical University Innsbruck, Anichstraße 35, A-6020 Innsbruck, Austria; 2 Department of Urology, Medical University Innsbruck, Anichstraße 35, A-6020 Innsbruck, Austria

**Keywords:** ^177^Lu-PSMA-617, Metastasized castration, resistant prostate cancer, Dosimetry, Post-therapy whole-body scintigraphy, ^68^Ga-PSMA-11, Theragnostic concept

## Abstract

**Introduction:**

A new therapeutic option for metastatic castration–resistant prostate cancer (mCRPC) of heavily pre-treated patients lies in ^177^Lu-PSMA-617 radioligand therapy.

**Methods:**

On the basis of PSMA-targeted ^68^Ga-PSMA-11 PET/CT, 32 consecutive mCRPC patients were selected for ^177^Lu-PSMA-617 therapy (6 GBq/cycle, 2 to 6 cycles, 6–10 weeks apart) and followed until death. Post-therapy whole-body (WB) dosimetry and ^68^Ga-PSMA-11 PET/CT data were compared and related to progression free and overall survival.

**Results:**

^177^Lu-PSMA-617 dosimetry after the first cycle indicated high tumor doses for skeletal (4.01 ± 2.64; range 1.10–13.00 Gy/GBq), lymph node (3.12 ± 2.07; range 0.70–8.70 Gy/GBq), and liver (2.97 ± 1.38; range 0.76–5.00 Gy/GBq) metastases whereas the dose for tissues/organs was acceptable in all patients for an intention-to-treat activity of 24 GBq. Any PSA decrease after the first cycle was found in 23/32 (72%), after the second cycle in 22/32 (69%), after the third cycle in 16/28 (57%), and after the fourth cycle in 8/18 (44%) patients. Post-therapy 24 h WB scintigraphy showed decreased tumor-to-background ratios in 24/32 (75%) after the first therapy cycle, after the second cycle in 17/29 (59%), and after the third cycle in 13/21 (62%) patients. The median PFS was 7 months and the median OS 12 months**.** In the group of PSA responders (*n* = 22) the median OS was 17 months versus 11 months in the group of non-responders (*n* = 10), *p* < 0.05. Decreasing SUV_max_ values were found for parotid (15.93 ± 6.23 versus 12.33 ± 4.07) and submandibular glands (17.65 ± 7.34 versus 13.12 ± 4.62) following treatment, along with transient (*n* = 6) or permanent (*n* = 2) xerostomia in 8/32 (25%) patients. In 3/32 patients, nephrotoxicity changed from Grade 2 to 3, whereas neither Grade 4 nephrotoxicity nor hematotoxicity was found. In most patients a good agreement was observed for the visual interpretation of the tracer accumulation between 24 h WB and PET/CT scans. However, no significance could be calculated for baseline-absorbed tumor doses and SUV_max_ values of tumor lesions. 5/32 (16%) patients showed a mixed response pattern, which resulted in disease progression over time.

**Conclusion:**

Serial PSA measurements and post-therapy 24 h WB scintigraphy seems to allow a sufficiently accurate follow-up of ^177^Lu-PSMA-617-treated mCRPC patients whereas ^68^Ga-PSMA-11 PET/CT should be performed for patient selection and final response assessment.

**Electronic supplementary material:**

The online version of this article (10.1007/s00259-019-04583-2) contains supplementary material, which is available to authorized users.

## Introduction

In recent years, the ^68^Ga-PSMA-labelled PSMA conjugate (Glu-NH-CO-NH-Lys-(Ahx)-[^68^Ga(HBED-CC)]) (PSMA-11) has successfully been used for lesion detection in case of biochemical recurrence as well as for improving primary staging in prostate cancer (PC) patients [1]. In accordance with the theragnostic concept, ^68^Ga-PSMA-11 PET/CT can be used to assess response to therapy with ^177^Lu-/^225^Ac-PSMA-617 in patients with metastatic castration–resistant PC (mCRPC). The currently most commonly used Reponse Evaluation Criteria In Solid Turmors (RECIST) [2] are based on cross-sectional abdominopelvic imaging together with bone scintigraphy and PSA serum levels. ^68^Ga-PSMA-11 PET/CT revealed a higher probability for a positive PET-finding in patients with low PSA values (≤ 0.5 ng/ml) than any other imaging modality including ^18^F-choline PET/CT [3], which can substantially influence the clinical management. In fact, ^68^Ga-PSMA-11 provides more accurate assessment than conventional imaging [4] and can also be superior to ^18^F-sodium fluoride PET/CT for the evaluation of bone metastases in response to ^223^Ra-chloride therapy [5].

In line with the theragnostic concept [6], ^68^Ga-labelled PSMA ligands are increasingly used to detect lesions in patients with mCRPC, prior to PSMA-directed radioligand therapy (PRLT), and as diagnostic response tool in the follow-up of PRLT-treated patients [1].

In this study, we used the modified PET Response Criteria In Solid Tumors (PERCIST, Visual and Semiquantitative “PET Score”) [7] and compared the results to the post-therapeutic 24 h whole-body (WB) scans (visual and semiquantitative “WB Score”) as well as to the PSA response (“PSA Score”) in 32 consecutive mCRPC patients undergoing serial ^177^Lu-PSMA-617 treatment. Progression free survival (PFS) and overall survival (OS) was correlated to each one of the scores, and different aspects of response assessment are discussed.

## Material and methods

### Ethical and regulatory issues

The application of ^177^Lu-PSMA-617 was approved by the institutional review tumor board and all patients gave written informed consent to therapy and imaging studies. All patients received ^177^Lu-PSMA-617 under compassionate use condition according to the updated Declaration of Helsinki [8], prepared according to the Austrian Medicinal Products Act, AMG §8 and §62. [9]. All patients were informed about the experimental nature of the ^177^Lu-PSMA-617 therapy and no systematic patient selection was performed. All regulations of the Austrian Agency for Radiation Protection were observed [10].

### Patient selection

All patients were selected on the basis of progressive mCRPC diagnosis based on ^68^Ga-PSMA-11 PET/CT imaging. The first 10 patients with mCRPC were prospectively assigned to undergo ^177^Lu-PSMA-617 therapy with three cycles (each 6 GBq) applied 8 to 10 weeks apart [11]. Four patients (Nos. 10, 11, 20, 28) received additional treatment activities which were not taken into account in the analyses presented herein. The following consecutive 22 patients were prospectively assigned to undergo ^177^Lu-PSMA-617 therapy with four cycles (each 6 GBq) applied 6 weeks apart as dosimetry had shown that higher doses are safe [11]. ^177^Lu-PSMA-617 was offered as surrogate therapy to patients who were refractory to chemotherapy, monoclonal antibody therapy, and/or hormonal therapy. Five patients were pre-treated with ^223^Ra-chloride and other four patients received zoledronic acid/alendronic acid as supportive therapy (Supplement 1 - Demographic data of metastatic castration resistant prostate cancer patients). In 8 patients (nos. 1, 3, 6, 7, 8, 14, 20, 32), bone-targeted therapy was continued after ^177^Lu-PSMA-617 therapy had commenced.

### Preparation of radio-labeled PSMA-targeting ligands

The GMP-precursors DOTA-PSMA-617 and PSMA-11 were obtained from Advanced Biochemical Compounds (ABX, Radeberg, Germany), no-carrier-added ^177^Lu-chloride (EndolucinBeta) from Isotope Technologies Garching GmbH (ITG, Garching, Germany), and ^68^Ga-chloride was obtained by elution of a ^68^Ge/^68^Ga generator (IGG100; Eckert & Ziegler, Berlin; 1.850 MBq reference activity) with 6 ml 0.1 N HCl. The preparation of ^177^Lu-PSMA-617 and of ^68^Ga-PSMA-11 was previously described in detail [11]. A fully automated synthesis module based on single-use cassettes (GalliaPharm; Eckert & Ziegler, Berlin; 1.850 MBq reference activity) was used.

### Administration of ^177^Lu-PSMA-617 and safety procedures

According to the Austrian Radiation Protection laws, all patients were treated as in-patients at the Nuclear Medicine ward and could be discharged 48 h post-injection. Clinical examinations were done prior to therapy and before discharge. Patients received an intravenous hydration (1000 ml 0.9% NaCl, flow 300 ml/h) starting 30 min prior to ^177^Lu-PSMA-617 therapy (flow 100 ml/h, 100 ml) which was administered by a dedicated infusion pump system. After each therapy cycle, blood cell count was determined every 2 weeks. In addition, every 4 weeks, renal and liver function parameters as well as PSA levels were evaluated. Laboratory values were classified into toxicity grades using the CTCAE, Common Terminology Criteria for Adverse Events 3.0. [12]. All patients were clinically monitored for vital parameters as well as possible side effects (such as xerostomia, nausea, vomiting, pain, tiredness, fatigue) using the standard hospital monitoring and documentation systems during their residence. Response criteria of the Eastern Cooperative Oncology Group (EOCG) performance status were used to assess quality of life (QoL). The ECOG status ranged from 0 to 1 (0 = fully active and 5 = dead).

### Response assessment

Morphological and functional imaging assessments were done by ^68^Ga-PSMA-11 PET/CT before the first PSMA-617 therapy cycle, and during follow-up. The study evaluation followed an intention-to-treat approach in all patients, and patients were followed up until death. PET scans were compared to whole-body (WB) scans acquired at 24 h post-infusion of ^177^Lu-PSMA-617 at baseline and during the follow-up period. Response was assessed following each treatment cycle. For response assessment, RECIST/PERCIST criteria and intensity of uptake (SUV_max_) in metastases in ^68^Ga-PSMA-11 PET/CT as well as tumor/background (TU/BG) ratios in ^177^Lu-PSMA-617 WB scans were used. Progressive disease (PD) was defined by appearance of new lesions and/or increase of uptake, partial remission (PR) by disappearance of one or more lesions and/or decrease of uptake, stable disease (SD) by no changes in number and uptake of the tumor lesions, and mixed response (MX) by disappearance and/or decrease of uptake of some lesions next to appearance of new lesions.

### ^68^Ga-PSMA-11 PET/CT imaging and SUV_max_ analysis—“PET Score”

^68^Ga-PSMA-11 PET/CT imaging was performed using a dedicated PET/CT system (Discovery 690; GE Healthcare, Milwaukee, WI) for patient selection and treatment response evaluation. An average activity of 150 MBq (range 120–160 MBq) ^68^Ga-PSMA-11 was administered intravenously. In all patients, an attenuation-corrected WB scan (skull to mid-thighs) in three-dimensional mode (emission time 2 min with an axial field-of-view of 15.6 cm per bed position) starting about 60 min after tracer injection was acquired with an image matrix size of 128 × 128 (pixel size 5.5 mm). In all patients, a low-dose CT scan was performed for attenuation correction of the PET emission data. The low-dose CT scan parameters using “GE smart mA dose modulation” were 100 kVp, 50 mA, 0.8 s per tube rotation, slice thickness 3.75 mm, and pitch 1.375.

All ^68^Ga-PSMA-11 PET/CT images were analyzed with dedicated commercially available software (eNTEGRA; GE Healthcare), which allowed the review of PET, CT, and fused imaging data. PET/CT images were interpreted by at least two board-certified nuclear medicine physicians with more than 5 years of clinical experience aware of all clinical data available. Visual interpretation was the main criterion for reaching the final diagnosis. Higher uptake as compared to surrounding BG activity, which did not correlate with physiological tracer uptake, was considered pathological and suspicious for malignancy. In addition, semiquantitative analysis of all pathological lesions was performed by comparing the maximum standardized uptake value (SUV_max_) in the 60-min scan with BG activity. All patients had multiple lesions (prostate bed, bone, lymph nodes, liver) which were chosen for SUV_max_ analysis. SUV_max_ calculation was obtained by drawing circular region of interests (ROIs) using eNTEGRA. Several areas of background were selected corresponding to the location of the pathological lesions.

### ^177^Lu-PSMA-617 WB, dosimetry calculation, and TU/BG ratios—“^177^Lu Score”

Dosimetry based on the MIRD principle was performed following the application of the first ^177^Lu-PSMA-617-therapy cycle. All patients received planar anterior and posterior WB scans with a dual-headed gamma camera (SIEMENS Symbia, Erlangen, Germany). For imaging, a medium energy parallel whole collimator was used, the scan speed was set to 15 cm/min, and a photo-peak window was centered at 113 keV and 208 keV with an energy window width of 15%. Scans were performed at about 0.5 h, 4 h, 24 h, 72 h, and 96 h post-infusion. In addition, SPECT/CT imaging of the abdomen was performed at 24 h to rule out possible overlays between different organs/tumors and to evaluate organ and tumor volumes. ROIs of tumors and all relevant organs at risk (OAR) were drawn on the 24 h image using the Hermes software. In addition, a ROI was drawn near the femur and one at the sinus frontalis to establish an appropriate background correction. All ROIs were copied to the other images (0.5 h, 4 h, 76 h, and 96 h) and the geometric mean of the anterior and posterior projections of the planar image was further analyzed by an Excel script. OLINDA/EXM-based [13] dosimetry was performed according to the information provided in the Supplement 2, Dosimetry Calculations.

To obtain the patient’s therapy outcome, we determined the “^177^Lu Score.” For each tumor lesion that was described in the WB dosimetry, as well as for salivary glands and the liver, ROIs were drawn to get the mean counts per pixel of a region divided by the respective value for a background region located in the thigh, thus resulting in TU/BG ratios. The WB dosimetry was performed alongside the first therapy cycle of each patient and for each following cycle a 24 h WB scan was acquired. The decrease in TU/BG ratios under therapy was calculated by normalizing the ratios to 100% (i.e., 1 for the first therapy).

### Statistics

Kaplan–Meier plotting was done for PFS and OS from initiation of ^177^Lu-PSMA-617 treatment with corresponding 95% confidence intervals (95% CI). Excel (Microsoft Office 2010) was used for Waterfall analysis of TU/BG and PSA changes. Correlations between changes of SUV_max_ values, PSA values, TU/BG ratios with PFS and OS, and initial absorbed dose calculations of tumor lesions were assessed by linear least squares regression. The coefficient of determination, R2, is the same as the Pearson correlation coefficient, *r*, squared. The Wilcoxon’s signed rank test was used to compare different groups. Furthermore, the Cox proportional hazard analysis was performed in order to evaluate the influence of different covariates on OS. A *p* value lower than 0.05 was considered statistically significant. All results are expressed as mean ± SD.

## Results

### Dosimetry

Distinct dosimetry data for ^177^Lu-PSMA-617 following the first treatment cycle in all consecutive patients are depicted in Table 1 for major healthy organs as well as skeletal, lymph node, and visceral metastases. In Table 2, the number of treatment cycles and the accumulated activities are shown together with the calculated SUV_max_ values at baseline and during the follow-up period. Basically, for skeletal metastases, lymph node, and visceral metastases, a large variation of the tumor dose was estimated for the various tumor lesions. The tables clearly indicate not only an inter-individual but also intra-individual variation for different metastases with calculated absorbed tumor doses up to 317 Gy for a single skeletal metastasis.

**Table 1:** Dosimetric calculations of ^177^Lu-PSMA6-17 therapy (Gy/GBq)

Patient	Red marrow	Lacrimal glands	Parotid glands	Submandibular glands	Kidneys	Urinary bladder wall	Osteogenic cells	Spleen	Liver	Small intestine	Gallbladder wall	Pancreas	ULI wall	LLI wall	Effective dose (mSv/MBq)	Skeletal metastases	Lymph node metastases	Visceral metastases
1	0.027	0.680	0.390	0.460	0.674	0.687	0.079	0.089	0.048	0.027	0.028	0.028	0.027	0.028	0.063	1.700	np	/
2	0.024	0.500	0.600	0.460	0.970	0.137	0.060	0.179	0.110	0.025	0.027	0.026	0.025	0.024	0.046	3.680	/	/
3	0.070	0.800	0.250	0.660	1.390	0.127	0.255	0.079	0.256	0.078	0.082	0.081	0.078	0.077	0.117	3.120	/	/
4	0.018	1.100	0.450	0.220	0.319	0.017	0.045	0.186	0.082	0.017	0.019	0.019	0.017	0.017	0.024	2.300	/	/
5	0.074	2.700	0.750	0.650	0.614	0.145	0.128	0.185	0.087	0.342	0.042	0.042	0.044	0.041	0.056	5.900	/	/
6	0.027	0.630	0.850	0.630	0.457	0.149	0.069	0.020	0.119	0.019	0.021	0.020	0.019	0.019	0.041	1.700	2.850	1.700
7	0.096	0.860	0.500	0.580	0.109	0.336	0.411	0.096	/	0.100	0.147	0.117	0.104	0.094	0.264	7.170	/	3.250
8	0.017	1.300	1.040	0.440	0.638	0.127	0.059	0.023	0.125	0.023	0.025	0.024	0.023	0.022	0.046	4.950	/	/
9	0.045	/	0.420	/	0.372	0.229	0.182	0.061	0.123	0.062	0.063	0.063	0.062	0.062	0.076	1.100	/	2.350
10	0.024	0.480	0.360	0.380	0.461	0.262	0.083	0.317	0.093	0.030	0.031	0.031	0.030	0.030	0.050	2.800	2.250	/
11	0.014	0.540	0.370	0.410	0.463	0.199	0.056	0.019	0.036	0.019	0.019	0.019	0.018	0.018	0.038	4.190	3.600	/
12	0.060	1.300	0.900	0.700	2.830	0.630	0.220	0.070	0.230	0.069	0.072	0.072	0.069	0.068	0.230	1.390	1.720	/
13	0.024	1.100	0.440	0.450	0.570	0.052	0.097	0.039	0.121	0.039	0.041	0.040	0.039	0.038	0.053	3.400	6.200	4.300
14	0.035	0.830	0.850	0.300	0.881	0.169	0.139	0.043	0.102	0.042	0.044	0.043	0.042	0.042	0.070	/	1.900	np
15	0.041	1.150	0.720	0.700	2.340	0.188	0.166	0.665	0.165	0.049	0.052	0.052	0.049	0.048	0.080	/	1.965	/
16	0.039	0.400	0.260	0.240	0.826	0.097	0.156	0.045	0.095	0.045	0.046	0.046	0.045	0.045	0.067	3.000	/	/
17	0.019	0.360	0.540	0.160	0.564	0.186	0.076	0.069	0.087	0.026	0.027	0.027	0.026	0.026	0.049	/	4.000	/
18	0.070	1.600	0.800	0.800	1.180	0.250	0.281	0.095	0.437	0.095	0.101	0.098	0.095	0.093	0.139	/	/	3.600
19	0.049	1.400	0.360	0.520	0.826	0.662	0.199	0.280	0.176	0.060	0.062	0.061	0.060	0.060	0.112	13.000	/	5.000
20	0.010	1.000	0.580	0.300	0.610	0.070	0.050	0.080	0.110	0.019	0.021	0.021	0.019	0.019	0.040	/	3.000	/
21	0.030	0.900	0.500	0.370	1.210	0.041	0.012	0.037	0.100	0.041	0.043	0.043	0.041	0.041	0.052	6.400	8.700	/
22	0.020	0.650	0.500	0.190	0.710	0.110	0.062	0.020	0.080	0.023	0.024	0.024	0.023	0.022	0.045	/	2.900	/
23	0.020	0.370	0.300	0.520	0.490	0.240	0.090	0.033	0.066	0.033	0.034	0.034	0.033	0.033	0.050	10.700	/	2.800
24	0.020	0.440	0.230	0.280	0.430	0.530	0.070	0.023	0.100	0.024	0.025	0.024	0.024	0.024	0.050	6.000	2.000	/
25	0.090	/	/	/	0.320	0.220	0.360	0.110	0.110	0.110	0.110	0.110	0.110	0.110	0.110	6.660	/	/
26	0.130	0.650	0.650	0.650	1.230	0.150	0.520	0.150	0.150	0.150	0.150	0.150	0.150	0.150	0.170	3.720	np	np
27	0.020	0.440	0.440	0.440	0.370	0.063	0.059	0.650	0.160	0.021	0.024	0.024	0.021	0.021	0.045	/	0.700	0.760
28	0.040	0.660	0.810	0.350	0.670	0.055	0.180	0.177	0.088	0.056	0.057	0.057	0.056	0.055	0.069	3.700	np	np
29	0.024	0.430	0.600	0.480	0.710	0.140	0.098	0.032	0.100	0.032	0.034	0.033	0.032	0.032	0.056	/	1.800	/
30	0.020	0.380	0.480	0.340	0.270	0.120	0.060	0.100	0.068	0.022	0.023	0.023	0.022	0.022	0.034	3.200	/	/
31	0.020	∕	0.400	0.650	0.640	0.150	0.080	0.030	0.070	0.026	0.027	0.027	0.026	0.026	0.048	0.800	6.300	/
32	0.030	∕	0.220	0.330	0.540	0.070	0.100	0.030	0.260	0.034	0.037	0.035	0.033	0.032	0.056	2.100	/	/
Min	0.010	0.360	0.220	0.160	0.109	0.017	0.012	0.019	0.036	0.017	0.019	0.019	0.017	0.017	0.024	0.800	0.700	0.760
Mean	0.039	0.845	0.534	0.455	0.771	0.207	0.141	0.126	0.128	0.055	0.049	0.047	0.046	0.045	0.076	4.278	3.326	2.970
SD	0.028	0.505	0.217	0.171	0.564	0.178	0.116	0.158	0.079	0.061	0.035	0.032	0.032	0.031	0.055	2.967	2.165	1.377
Max	0.130	2.700	1.040	0.800	2.830	0.687	0.520	0.665	0.437	0.342	0.150	0.150	0.150	0.150	0.264	13.000	8.700	5.000

For skeletal, lymphnode, and visceral metastases, the mean values are stated if the dose for several metastases could be calculated

*np* = not possible to calculate

/ = not present

**Table 2:** Dosimetric calculations of ^177^Lu-PSMA-617 therapy compared to SUV_max_ values

Skeletal metastases						Lymph node metastases						Visceral metastases					
Absorbed dose			SUV_max_			Absorbed dose			SUV_max_			Absorbed dose			SUV_max_		
Patient	Therapy cycles	Accumulated activity (GBq)	D/A^a^(Gy/GBq)	Dtot^b^(Gy)	Baseline	After 2 cycles	After 3/4 cycles	D/A1(Gy/GBq)	Dtot2 (Gy)	Baseline	After 2 cycles	After 3/4 cycles	D/A1(Gy/GBq)	Dtot2 (Gy)	Baseline	After 2 cycles	After 3/4 cycles
1	3	17.7	1.70	30.16	12.40	9.10	7.20	np	np	16.50	3.33	7.93	/	/	/	/	/
2	3	19.3	3.68	71.17	21.53	8.40	/	/	/	/	/	/	/	/	/	/	/
3	3	18.6	3.12	58.13	18.12	11.00	11.97	np	np	20.88	4.60	2.95	/	/	/	/	/
4	3	16.3	2.30	37.49	8.00	5.40	5.49	/	/	/	/	/	/	/	/	/	/
5	3	17.9	5.90	105.49	15.00	6.40	8.70	/	/	4.10	1.20	1.10	/	/	/	/	/
6	3	18.1	1.70	30.80	30.37	28.87	22.07	2.85	51.64	16.00	21.80	22.50	1.70	30.80	28.55	34.25	24.70
7	3	18.8	7.50	141.23	27.65	18.83	14.10	/	/	/	/	/	3.25	61.20	16.90	12.20	14.60
8	2	12.7	4.95	62.87	9.99	12.79	/	/	/	/	/	/	/	/	/	/	/
9	3	18.6	1.10	20.45	28.93	15.63	/	/	/	/	/	/	2.35	43.69	24.10	17.75	/
10	4 + 2	24.3	2.80	68.04	18.99	13.32	9.21	2.25	54.68	21.43	12.48	np	/	/	/	/	/
11	4 + 2	25.6	4.19	107.31	7.10	15.10	10.30	3.60	92.20	66.40	4.80	8.80	/	/	/	/	/
12	2	12.8	1.39	17.78	15.00	7.10	/	1.72	22.00	16.70	8.00	/	/	/	/	/	/
13	4	25.3	3.40	86.09	22.10	/	12.50	6.20	156.98	66.30	/	29.30	4.30	108.88	37.90	/	15.40
14	4	24.9	/	/	/	/	/	1.90	47.31	18.20	/	/	np	np	2.50	/	/
15	3	18.3	/	/	11.20	21.40	6.80	2.08	38.04	10.20	28.70	19.60	/	/	/	/	/
16	4	23.3	4.00	93.36	31.00	/	/	/	/	/	/	/	/	/	/	/	/
17	4	24.9	/	/	/	/	/	4.00	99.60	65.20	/	16.10	/	/	/	/	/
18	3	18.1	/	/	/	/	/	/	/	/	/	/	3.60	65.16	19.30	/	14.00
19	4	24.4	13.00	317.20	37.50	/	4.80	/	/	/	/	/	5.00	122.00	14.15	12.20	10.85
20	4 + 2	37.13	/	/	/	/	/	3.00	111.39	21.60	/	8.60	/	/	/	/	/
21	2	10.8	6.40	68.86	22.40	20.10	/	8.70	93.61	106.60	61.40	/	/	/	/	/	/
22	4	23.8	/	/	/	/	/	2.90	69.02	39.80	5.40	3.20	/	/	/	/	/
23	4	24.6	2.37	58.30	57.27	/	16.30	/	/	/	/	/	2.80	68.88	22.66	/	17.31
24	4	25.3	6.00	151.62	5.00	/	3.40	2.00	50.60	10.00	/	7.80	/	/	/	/	/
25	4	24.4	6.66	162.37	36.40	/	/	/	/	37.80	/	/	/	/	/	/	/
26	4	25.3	3.72	94.26	29.20	/	2.20	np	np	15.90	/	3.30	np	np	17.30	/	11.80
27	3	19.0	/	/	/	/	/	0.70	13.30	15.90	/	/	0.76	14.44	16.50	/	/
28	4 + 1	25.0	3.70	92.50	15.70	/	12.00	np	np	10.13	/	5.57	np	np	/	/	/
29	3	16.0	/	/	/	/	/	1.80	28.87	18.90	/	24.20	/	/	/	/	/
30	4	25.5	3.20	81.60	12.79	/	3.35	np	np	9.25	/	1.21	/	/	/	/	/
31	4	23.7	1.25	18.96	41.06	/	7.70	6.30	149.31	44.15	/	14.54	/	/	/	/	/
32	4	21.5	2.10	45.15	26.95	/	10.16	/	/	/	/	/	/	/	/	/	/
Mean		21.3	4.01	89.31	22.47	13.82	9.80	3.12	66.37	29.93	15.17	11.50	2.97	64.38	19.99	19.10	15.52
SD		5.2	2.64	66.26	12.36	6.71	5.26	2.07	39.92	26.36	18.48	9.12	1.38	36.61	9.35	10.43	4.59
Minimum		10.8	1.10	17.78	5.00	5.40	2.20	0.70	13.30	4.10	1.20	1.10	0.76	14.44	2.50	12.20	10.85
Maximum		37.1	13.00	317.20	57.27	28.87	22.07	8.70	156.98	106.60	61.40	29.30	5.00	122.00	37.90	34.25	24.70
^a^absorbed dose per unit administered activity	Max/min	3.5	11.82	17.84	11.45	5.35	10.03	12.43	11.80	26.00	51.17	26.64	6.58	8.45	15.16	2.81	2.28

^b^total cumulative dose

*np* = not possible to calculate

/ = not performed

The accumulated administered activity of ^177^Lu-PSMA-617 in 32 patients receiving 2 to 6 cycles was 21.3 ± 5.2 GBq (range 10.8–37.1 GBq). No relevant difference was observed for normal organ dosimetry in patients with either low or high tumor load. The mean effective dose was 0.076 ± 0.055 Sv/GBq (range 0.024–0.264 Sv/GBq). The mean absorbed dose for bone 4.4278 ± 2.967 (range 0.8–13.0) Gy/GBq, lymph node 3.326 ± 2.165 (range 0.7–8.7) Gy/GBq, and visceral lesions (2.970 ± 1.377, range 0.76–5.0 Gy/GBq) metastases was remarkably higher than for normal organs.

### Response evaluation by ^68^Ga-PSMA-11 PET/CT

**Fig. 1:**
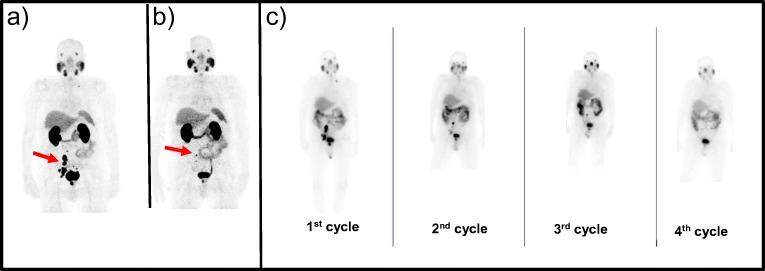
**Response evaluation of PET/CT and post-therapy whole-body scintigraphy**. ^68^Ga-PSMA-11 PET/CT of patient no. 17 before ^177^Lu-PSMA-617-therapy (**a**) and after four (**b**) cycles (24.9 GBq), and serial ^177^Lu-PSMA-617 24-h whole-body scans (**c**). In this patient, a representative SUV_max_ value of lymph nodes at baseline was 65.2 which decreased to 16.1 after 4 cycles and corresponded to decreasing TU/BG ratio of 25.2 at the first therapy cycle and 3.1 at the 4th therapy cycle. PSA dropped from 76.9 to 1.27

All patients were selected for treatment by positive ^68^Ga-PSMA-11 PET/CT scans. In all patients, evaluation of the WB PET scans was done by visual interpretation as well as by calculation of SUV_max_ at baseline and during the follow-up period (Table 2, Fig. 1).

#### “Visual PET Score”

Response to therapy in terms of decreased uptake of all visible lesions (“Visual PET Score”) was seen in 11/13 (84.62%) patients after the first therapy cycle (i.e. at the time of the second cycle which was 6 to 9 weeks after the first cycle, Table 3). After the second cycle 4/10 (40%) and after the third cycle 12/15 (80%), patients showed decreased uptake in all visible lesions. Increased uptake in terms of PD was seen in 1/13 (7.69%) patients after the first cycle, in 4/10 (40%) after the second cycle, and in 2/15 (13.33%) after the third cycle. In 1/13 (7.69%) patients, stable uptake in terms of SD was seen after the first cycle, in 2/10 (20%) patients after the second cycle, and in 1/15 (6.67%) patients after the third cycle.

**Table 3:** Overall response evaluation to ^177^ Lu-PSMA-617 therapy

Patient	Visual whole-body score Therapy cycle			Lu Score			Visual PET Score				PSA Score				OS	PFS
				Therapy cycle			Therapy cycle				Therapy cycle					
	2.	3.	4.	2.	3.	4.	2.	3.	4.	FU	2	3.	4.	FU	Months	Months
1	↑	↑		↑	↑			↑			↑	↑	↓		10	4
2	↓	↓	↓	↓	↓	↓			↓		↓	↓	↑	↑	7	7
3	↕	↓	↑	↓	↕	↕	↓	↑			↓	↓	↑		12	4.5
4	↓	↑		↓	↓		↓	↑			↓	↓	↑		11	4.5
5	↑	↓	↓	↓	↑	↓	↓	↔			↑	↓	↓	↑	16	13
6	↑	↓	↓	↓	↕	↓	↔	↔	↓		↓	↑	↓	↓	9	4
7	↕	↓		↕	↓		↓	↓			↓	↑	↑		11	7
8	↑			↓			↑				↑	↑			9	4
9	↑	↓		↓	↑		↓	↓			↑	↑	↓		19	5
10	↓	↑	↓	↓	↕	↓	↓		↓	↑	↑	↓	↓	↑	18	12
11	↓	↑	↓	↓	↓	↓			↓		↓	↓	↓	↓	31	13
12	↑			↑			↓				↓	↑			12	5
13	↓	↑	↑	↓	↓	↑			↑		↓	↓	↓	↓	14	8
14	↓	↑	↓	↓	↑	↔					↓	↓	↓	↓	16	Not known
15	↓	↓		↓	↓		↓				↓	↓	↓		25	Not known
16	↓	↔	↕	↔	↓	↑					↓	↓	↑		5	2,5
17	↓	↓	↓	↓	↓	↓			↓		↓	↓	↓	↓	Alive	Stable disease
18	↓	↔		↓	↓		↓				↓	↓	↑		5	4
19	↓	↓	↓	↓	↓	↓			↓		↑	↓	↑	↑	9	6
20	↓	↓	↑	↔	↓	↓	↓		↓	↓	↓	↓	↓	↔	Alive	24
21	↓			↓			↓				↓	↓			11	3
22	↓	↓	↓	↓	↓	↓	↓		↓	↓	↓	↓	↓	↓	Alive	18
23	↑	↑	↓	↔	↔	↔			↓		↓	↑	↑	↓	9	3
24	↑	↓	↔	↑	↓	↓			↔		↑	↑	↓	↑	9	6
25	↓	↓	↕	↓	↔	↓					↓	↓	↓		11	6
26	↓	↓	↓	↓	↓	↓			↓	↑	↓	↓	↓	↓	Alive	6
27	↑	↑		↑	↑						↑	↑			5	3
28	↓	↓	↑	↓	↔	↑		↓			↓	↓	↑	↑	11	7
29	↓	↕		↓	↓			↑			↓	↓	↑		8	5
30	↔	↓	↑	↓	↓	↑			↓	↕	↑	↑	↓	↑	14	7
31	↔	↓	↕	↓	↓	↓			↓	↓	↓	↓	↑	↑	Alive	Stable disease
32	↓	↑	↑	↓	↑	↑			↑		↓	↓	↑	↑	Alive	6
↓ = therapy response	19	17	11	24	17	13	11	4	12	3	23	22	16	8		
↑ = progressive disease	9	9	6	4	6	5	1	4	2	2	9	10	12	9		
↔ = stable disease	2	2	1	3	3	2	1	2	1					1		
↕ = mixed response	2	1	3	1	3	1				1						
Patients’ total	32	30	21	32	29	21	13	10	15	6	32	32	28	18		

An upward arrow (↑ = progressive disease) was drawn when either the visual uptake in the tumor lesions at 24 h post-injection (whole-body “Visual Score”), the TU/BG ratios at 24 h post-injection (“Lu Score”), the visual uptake in the tumor lesions in the ^68^Ga-PET/CT follow-up scans (“PET Score”) of all tumor lesions, or the PSA levels (“PSA Score”) were increasing from one therapy cycle to the other. A downward arrow (↓ = therapy response) was drawn when respective values were decreasing. In case of a mixed response, a down/upward arrow (↕ ,i.e., disappearance of tumor lesions at one location and development of new lesions in a different location) and for stable disease, a right/left arrow was used (↔). FU, follow-up. In all patients, ^68^Ga-PSMA-11 PET/CT was performed for treatment selection (i.e., baseline) and was repeated after 2 to 6 therapy cycles

#### “Semiquantitative PET Score”—SUV_max_ calculations

Baseline SUV_max_ values of 22.47 ± 12.36 (range 5.0–57.27) decreased to 13.82 ± 6.71 (range 5.4–28.87) after 2 cycles and to 9.80 ± 5.26 (range 2.2–22.07) after 3 or 4 cycles in skeletal metastases (Table 2). Also SUV_max_ values of LN metastases decreased from 29.93 ± 26.36 (range 4.1–106.6) to 15.17 ± 18.48 (range 1.2–61.40) after the second cycle and to 11.5 ± 9.12 (range 1.1–29.30) after 3 or 4 cycles. SUV_max_ values calculated for visceral metastases decreased from 19.99 ± 9.35 (range 2.5–37.90) to 19.10 ± 10.43 (range 12.2–34.25) after 2 cycles and to 15.52 ± 4.59 (range 10.85–24.70) after 3 or 4 cycles.

### Response evaluation by ^177^Lu-PSMA-617 post-therapy 24 h WB scintigraphy

In all patients, evaluation of the 24 h WB scans was done by visual interpretation as well as by calculation of TU/BG ratios.

#### “Visual WB Score”

Response to therapy in terms of decreased uptake of all visible lesions (“WB Visual Score”) was seen in 19/32 (59.37%) patients after the first therapy cycle (i.e. at the time of the second cycle which was 6 to 9 weeks after the first cycle). After the second cycle 18/29 (62.07%) and after the third cycle 11/21 (52.38%) patients showed decreased uptake in all visible lesions (Table 3). Increased uptake in terms of PD was seen in 9/32 (28.13%) patients after the first cycle, in 9/29 (31.03%) after the 2nd cycle, and in 6/21 (28.57%) after the third cycle. In 2/32 (6.25%) patients, stable uptake was seen after the 1st cycle, in 2/29 (6.90%) patients after the 2nd cycle, and in 1/21 (4.76%) patients after the 3rd cycle. In 2/32 (6.25%) patients, a MX was observed after the first cycle and in 3/21 (14.29%) patients after the 3rd cycle.

#### “^177^Lu Score”

**Fig. 2:**
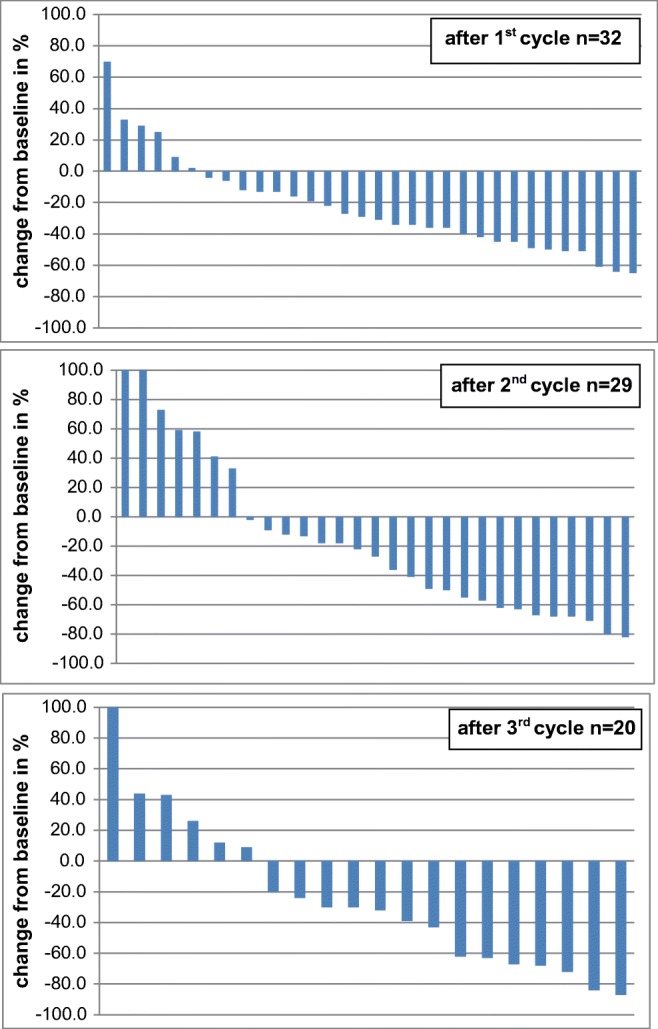
“^177^Lu Score”: response of TU/BG ratios calculated from the 24-h whole-body scans (waterfall plots)

Response to therapy in terms of decreased TU/BG ratios (“^177^Lu Score”) was seen in 24/32 (75%) patients after the 1st therapy cycle (i.e., at the time of the 2nd cycle which was 6 to 9 weeks after the 1st cycle). After the 2nd cycle 17/29 (58.62%) and after the 3rd cycle 13/21 (61.90%) patients showed decreased uptake in all visible lesions (Table 3, Fig. 2). Increased TU/BG ratios in terms of disease progression were seen in 4/32 (12.5%) patients after the 1st cycle, in 6/29 (20.68%) after the 2nd cycle, and in 5/21 (23.80%) after the 3rd cycle. In 3/32 (9.38%) patients, stable TU/BG ratios were seen after the 1st cycle, in 3/29 (10.34%) patients after the 2nd cycle, and in 2/21 (9.52%) patients after the 3rd cycle. In 1/32 (3.13%) patients, a MX was observed after the 1st cycle, in 3/29 (10.34%) patients after the 2nd cycle, and in 1/21 (4.76%) after the 3rd cycle.

### Comparison of response evaluation by ^68^Ga-PSMA-11 PET/CT and ^177^ Lu-PSMA-617 post-therapy 24 h WB scintigraphy

#### Visual interpretation

In most patients, visual interpretation of tracer accumulation in all lesions compared well between ^68^Ga-PSMA-11 PET/CT and 24 h post-therapy WB scintigraphy. At the time of the 4th therapy cycle, comparative data were available in 15 patients showing response in 12/15 (80%) patients by PET/CT and in 11/21 (52.38%) by 24 h WB scintigraphy.

#### SUV_max_ and absorbed tumor dose at baseline

**Fig. 3:**
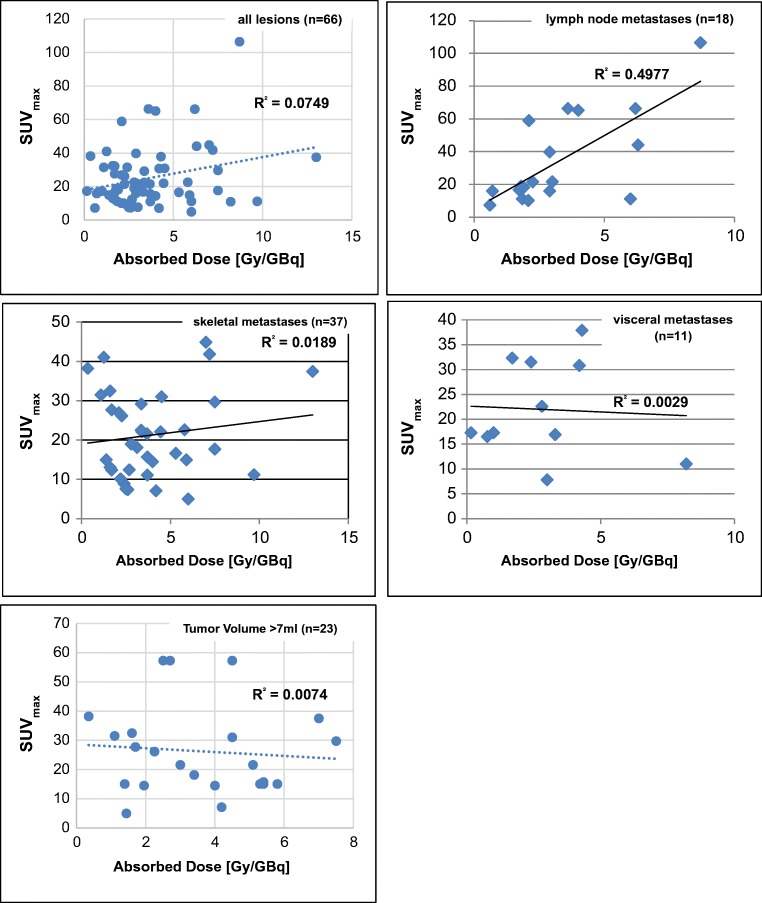
Correlation of SUV_max_ values with absorbed tumor dose

SUV_max_ values of tumor lesions at baseline were compared to calculated absorbed doses. Only tumor lesions that could accurately be defined on the 24 h WB post-therapeutic scans were taken for comparative analyses (Fig. 3). Whereas no correlation was found for all metastases (*R*^2^ = 0.0749), a weak correlation was found only for LN metastases (*R*^2^ = 0.4977). Also for skeletal (*R*^2^ = 0.0189) and for visceral metastases, no correlation could be calculated (*R*^2^ = 0.0029). Also for tumor lesions with an estimated volume > 7 ml, no correlation could be calculated *R*^2^ = 0.0074).

### PSA response—“PSA Score”

**Fig. 4:**
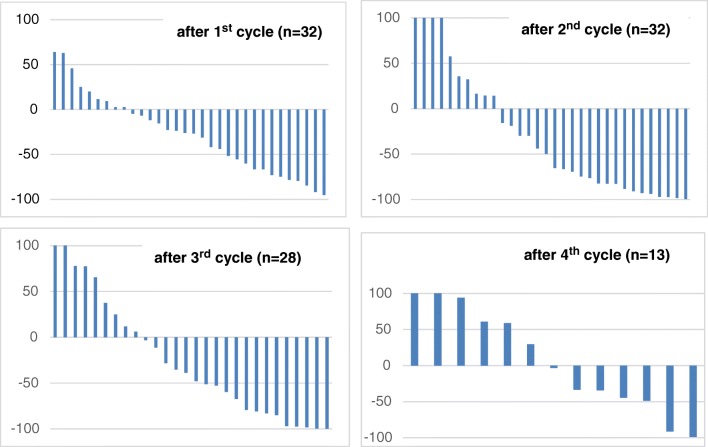
“PSA Score”: response of PSA following ^177^Lu-PSMA-617 treatment (waterfall plots)

PSA response to therapy is depicted in Fig. 4 and Table 3. After the first cycle, response > 50% was seen in 12/32 (37.5%) patients, after the second in 16/32 (50%) patients, after the third in 13/28 (46.43%) patients, and after the fourth cycle in 2/13 (15.38%) patients.

Any PSA response was seen in 23/32 (71.88%) patients after the first cycle, in 22/32 (68.75%) patients after the second cycle, in 16/28 (57.14%) patients after the third cycle, and in 8/18 (44.44%) patients in the follow-up.

In 5 patients (nos. 3, 16, 25, 30, and 31; Table 3) with MX response, the PSA values increased in the follow-up period.

### Safety evaluation and side effects

^177^Lu-PSMA-617 therapy was well tolerated by all patients. In none of the patients, significant adverse effects were reported during their hospitalization. In none of the patients, therapy-related Grade 4 side effects were observed regarding hemtotoxicity, hepatotoxicity, and nephrotoxicity. Change from Grade 1/2 to Grade 3 nephrotoxcity were observed in 3 patients, i.e., 10% (Table 4).

**Table 4:** Side effects of ^177^Lu-PSMA-617 treatment

Patient	Accumulated activity (GBq)		Number of cycles		Therapy-related side effects	
Hematoxicity (Grade)	Hepatotoxicity (Grade)		Nephrotoxicity (Grade)		Xerostomia	
1	17.7	3	I → I	I → I	0	0
2	19.3	4	0	I → I	I → I	0
3	18.6	4	I → I	I → I	0	0
4	16.3	3	0	0	0	Transient
5	17.9	4	I → I	I → I	0	Permanent
6	18.1	4	0	I → I	0	0
7	18.8	3	0	I → I	0	0
8	12.7	2	I → I	I → I	I → II	Transient
9	18.6	3	II → II	II → II	0	0
10	24.32 + 11.84	4 + 2	0	I → I	0	0
11	25.61 + 12.52	4 + 2	0	0	0	0
12	12.79	2	I → I	0	II → III	Transient
13	25.32	4	I → I	I → II	0	0
14	24.9	4	I → I	0	II → II	Transient
15	18.29	3	I → I	0	I → I	0
16	23.34	4	0	II → II	0	0
17	24.93	4	0	0	I → I	0
18	18.1	3	I → I	0	0	Transient
19	24.4	4	I → I	0	I → I	Permanent
20	24.8 + 12.33	4 + 2	0	0	0	0
21	10.76	2	I → I	0	II → II	0
22	23.84	4	0	0	I → I	0
23	24.61	4	0 →0	0	0	0
24	25.27	4	I →I	I →II	0	0
25	24.38	4	I→I	0→0	0→I	0
26	25.34	4	I→I	0→0	0→I	0
27	19	3	0→I	0→II	0→0	0
28	21.95 + 3.05	4 + 1	0→I	II→II	II→III	0
29	16.04	3	I→I	0→I	I→III	0
30	25.54	4	I → I	0 → 0	0 → 0	0
31	23.74	4	I → I	0 → 0	0 → 0	0
32	21.49	4	I → I	0 → I	I → I	Transient

Transient xerostomia was observed in 6 patients (i.e., 18.75%) and permanent xerostomia in 2 patients (6.4%) despite of no correlation with absorbed dose in the parotide or submandibular glands which both showed decreased SUV_max_ values and volumes after treatment in the majority of patients (Table 5).

**Table 5:** Dosimetric calculations of the salivary glands after ^177^Lu-PSMA-617 therapy (SUV_max_, volume)

Parotid glands							Submandibular glands							
Absorbed dose		Baseline			After 2–4 cycles		Absorbed dose			Baseline		After 2–4 cycles		
Patient	Therapy cycles	Accumulated activity (GBq)	D/A^a^(Gy/GBq)	Dtot^b^ (Gy)	SUV_max_	Volume (ml)	SUV_max_	Volume (ml)	D/A1(Gy/GBq)	Dtot2 (Gy)	SUV_max_	Volume (ml)	SUV_max_	Volume (ml)
1	3	17.74	0.39	6.92	26.00	27.00	15.45	21.40	0.46	8.16	19.65	8.65	11.45	8.00
2	4	19.34	0.60	11.60	14.40	29.23	10.55	22.25	0.46	8.90	16.80	7.57	11.35	6.10
3	4	18.63	0.25	4.66	9.35	24.65	8.45	21.00	0.66	12.30	12.45	9.05	13.85	6.85
4	3	16.30	0.45	7.34	10.25	18.75	10.20	14.95	0.22	3.59	10.35	9.40	10.65	9.10
5	4	17.88	0.75	13.41	7.85	25.75	10.05	19.80	0.65	11.62	8.35	7.45	10.25	6.15
6	4	18.12	0.85	15.40	17.80	22.35	10.35	21.50	0.63	11.42	23.15	9.85	14.20	8.20
7	3	18.83	0.50	9.42	14.95	18.90	16.95	15.80	0.58	10.92	17.50	9.30	18.25	8.05
8	2	12.70	1.04	13.21	7.50	34.00	7.00	26.00	0.44	5.59	9.05	7.30	7.45	6.10
9	3	18.59	0.42	7.81	8.05	25.70	8.75	17.30	/	/	7.00	7.35	4.75	7.25
10	4 + 2	24.30	0.36	8.75	7.80	23.50	18.70	29.00	0.38	9.23	9.40	10.35	17.50	11.95
11	4 + 2	25.61	0.37	9.47	18.50	20.39	15.65	21.95	0.41	10.50	16.60	8.00	13.40	7.62
12	2	12.79	0.90	11.51	17.10	18.53	9.35	22.34	0.70	8.95	17.10	11.15	7.70	12.51
13	4	25.32	0.44	11.14	26.45	21.86	11.10	13.35	0.45	11.39	28.40	9.49	13.30	6.16
14	4	24.90	0.85	21.16	16.20	56.10	/	/	0.30	7.47	19.30	10.16	/	/
15	3	18.29	0.72	13.16	10.80	16.79	8.65	18.62	0.70	12.80	15.75	7.25	14.75	5.68
16	4	23.34	0.26	4.70	13.20	20.24	/	/	0.24	4.34	13.65	7.53	/	/
17	4	24.93	0.54	/	14.70	15.64	19.35	24.15	0.16	0.97	7.75	1.85	8.22	6.65
18	3	18.10	0.80	14.48	13.10	14.13	9.35	8.16	0.80	14.48	12.55	8.36	6.20	6.35
19	4	24.40	0.36	6.76	13.85	18.82	8.55	13.74	0.52	9.77	18.65	8.31	8.30	5.77
20	4	24.80	0.58	14.38	24.30	14.35	16.95	12.52	0.30	7.44	22.90	13.02	18.70	9.10
21	2	10.76	0.50	5.38	10.90	21.66	16.60	22.05	0.37	3.98	13.00	8.31	16.15	8.50
22	4	23.80	0.50	11.90	16.50	30.66	10.85	24.16	0.19	4.52	20.75	8.75	14.15	7.82
23	4	24.61	0.30	7.38	17.26	20.70	11.28	20.10	0.52	12.79	24.87	8.13	14.58	7.68
24	4	25.27	0.23	5.81	/	/	/	/	0.28	7.07	/	/	/	/
25	4	24.40	/	/	15.25	21.66	/	/	/	/	20.75	4.88	/	/
26	4	25.34	0.65	16.47	10.15	17.26	10.75	10.82	0.65	16.47	5.90	4.64	10.90	5.43
27	3	19.00	0.44	8.36	13.15	29.19	/	/	0.44	8.36	18.35	10.17	/	/
28	4 + 1	25.00	0.81	20.25	15.45	28.94	22.00	26.06	0.35	8.75	19.85	11.44	19.85	10.56
29	3	16.04	0.60	9.62	23.70	16.77	15.90	14.03	0.50	8.02	22.15	6.50	19.00	4.93
30	4 + 1	25.54	0.48	12.29	29.69	17.18	12.49	12.91	0.34	8.68	33.93	8.66	19.67	7.62
31	4	23.74	0.40	9.49	23.20	22.09	9.35	21.21	0.65	15.43	28.41	8.27	21.21	9.66
32	4	21.49	0.22	4.72	26.37	13.97	8.20	11.63	0.33	7.09	32.75	5.91	8.40	5.57
Mean	20.93	0.53	10.56	15.93	22.80	12.33	18.77	0.46	9.03	17.65	8.29	13.12	7.61	
SD	4.33	0.22	4.33	6.23	8.07	4.07	5.35	0.17	3.61	7.34	2.15	4.62	1.93	
Min	10.76	0.22	4.66	7.50	13.97	7.00	8.16	0.16	0.97	5.90	1.85	4.75	4.93	
Max	25.61	1.04	21.16	29.69	56.10	22.00	29.00	0.80	16.47	33.93	13.02	21.21	12.51	
Max/min	2.38	4.73	4.54	3.96	4.02	3.14	3.55	5.00	16.98	5.75	7.04	4.47	2.54	

^a^absorbed dose per unit administered activity

^b^total cumulative dose

The ECOG status ranged from 0 to 1 (0 = fully active and 5 = dead) before therapy and only in 1 patient increased to 2 under therapy due to bone pain (for ECOG evaluation see Supplement 3).

### Survival data

**Fig. 5:**
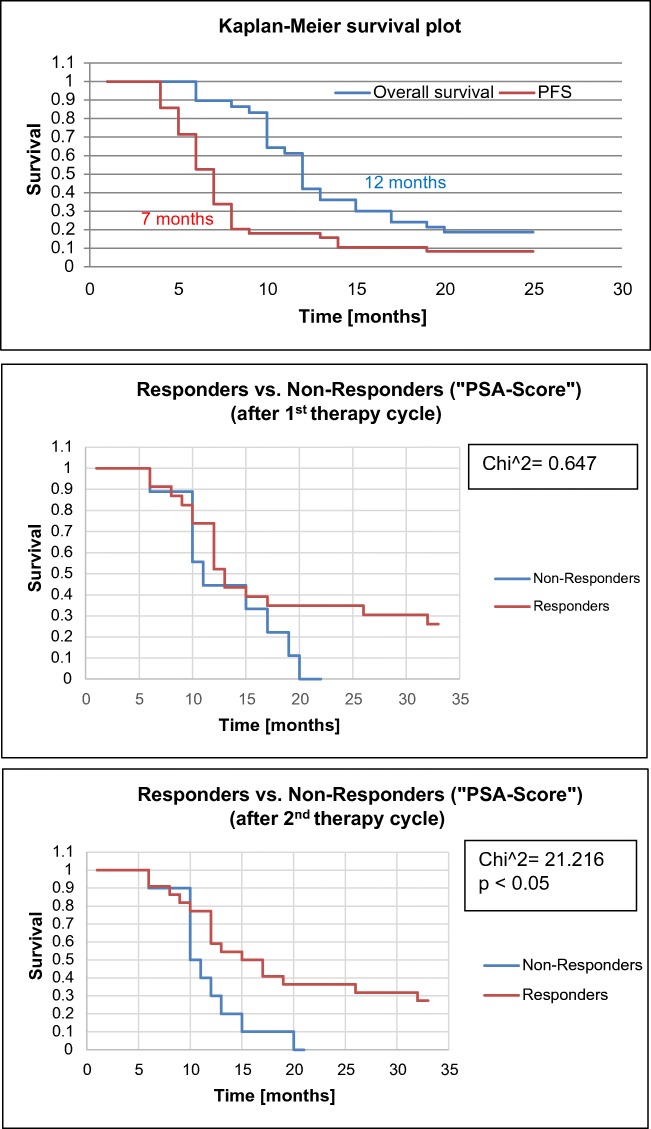
Kaplan–Meier curves for PFS and OS from the start of treatment

**Fig. 6:**
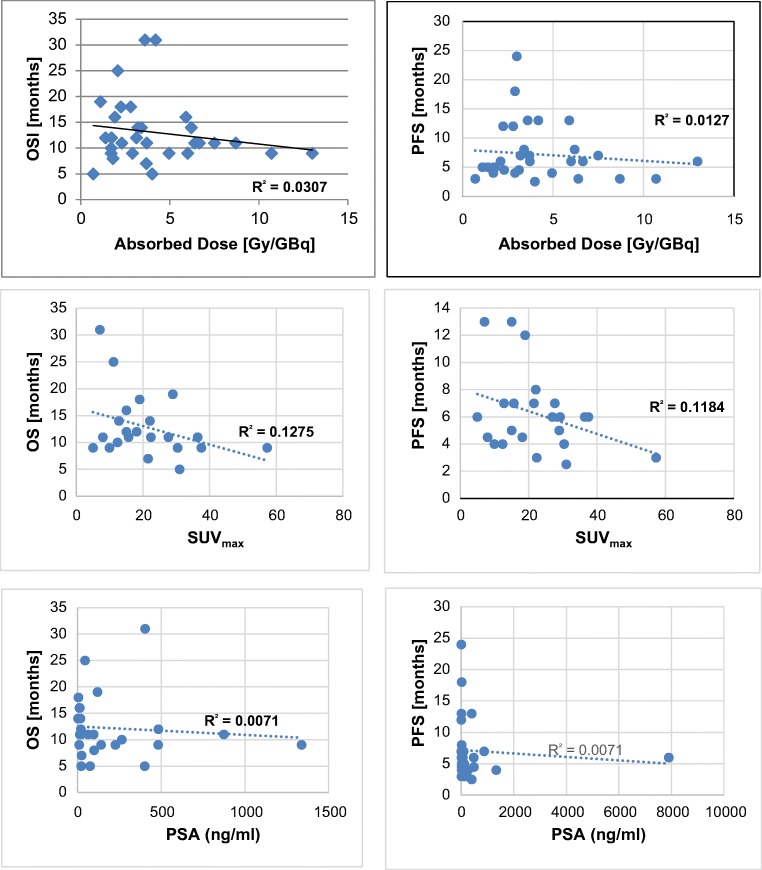
Correlation of PFS and OS with absorbed dose, SUV_max_, and PSA values

The median PFS was 7 months and the median OS was 12 months for all patients *n* = 32 (Fig. 5). The “PSA responders” for survival analysis were defined as patients showing any PSA decline in response to therapy. A significantly (*p* < 0.05) longer survival time could be calculated for responders versus non-responders following the second therapy cycle after an accumulated dose of 12 GBq of ^177^Lu-PSMA-617. In the group of responders (*n* = 22), the median survival time was 11 months versus 17 months in the group of non-responders (*n* = 10). However, no correlation was found for neither PFS (*R*^2^ = 0.0071) nor OS (*R*^2^ = 0.0071) with PSA levels at baseline (i.e., before treatment). Furthermore, no correlation was found for neither PFS (*R*^2^ = 0.0127) nor OS (*R*^2^ = 0.0307) with absorbed tumor doses and SUV_max_ values at baseline (Fig. 6). Additional statistical information can be derived in Supplement 4 and 5.

## Discussion

We report on a single-center study in a total of 32 consecutive mCRPC patients treated with ^177^Lu-PSMA-617. All patients were heavily pre-treated and were followed until death. mCRPC patients received up to 6 therapy cycles, 6 GBq each, 6 to 10 weeks apart. In all patients, visual interpretation and semiquantitative calculation of PET/CT (SUV_max_, i.e., “PET Score”) and 24 h WB scans (TU/BG ratios, i.e., “^177^Lu Score”) were performed and compared with PSA response over the course of disease. The median PFS was 7 months, the median OS was 12 months, and responders to 2 × 6 GBq therapy lived significantly (*p* < 0.05) longer than non-responders, i.e., 6 months (Fig. 5).

The response evaluation in terms of CR, PR, SD, or PD is usually assessed by biochemical response and metabolic/radiologic response based on modified PERCIST criteria [7]. However, the reported follow-up duration of the ^177^Lu-PSMA-617-treated patients in the literature varies between the different centers and together with different treatment regimes (i.e., treatment dose, number of treatment cycles, time span between the cycles, response evaluation), an appropriate comparison of treatment results is rather difficult. Therefore, published data are usually single-center studies, and moreover, only retrospective ones in most cases [1]. Some patients may also show a MX response which we have defined as disappearance of tumor lesions at one location and development of new lesions in at least one different location. In our study, in about 15% of patients a MX response was observed in both “Visual and Semiquantitative WB Score (i.e., “^177^Lu Score”)” as well as “PET Score” (i.e., SUV_max_). This observation of MX response underlines the well-known development of heterogeneity of PC lesions over time. Textural heterogeneity parameters may also play an important role explaining these findings [14]. In fact, all our patients with MX response also showed increasing PSA values over time und thus disease progression.

In the majority (approximately 2/3) of patients, the response in terms of decreasing “^177^Lu Score“ was well correlating with the “Visual 24 h WB Score” as well as “Visual and Semiquantitative (SUV_max_) “PET Score,” and the “PSA Score.” Following the first therapy cycle with 6 GBq of ^177^Lu-PSMA-617, 19/32 (60%) patients showed decreased visible tracer uptake in all lesions in the WB scintigraphy, in 24/32 (75%) patients in the “^177^Lu Score“, in 11/13 (85%) patients in all visible lesions in the ^68^Ga-PSMA-11 PET/CT, and in 23/32 (72%) patients in the “PSA Score” (i.e., biochemical response).

Whereas most responding patients had significant improvement already after the first treatment cycle with 6 GBq (only), in roughly 15% of patients, a delayed response after the second or more treatment cycles with additional 6 GBq each (accumulated dose 12 to 24 GBq) was observed by the “PSA Score” in 5/28 (16%) patients and in the “^177^Lu Score” in 4/29 (14%) patients and in 11/13 (85%) patients by the “PET Score.” Such a delayed response in patients who did not respond to the first cycle was also communicated earlier by Rahbar et al. [15] in 12/41 (29%) patients. This observation is of great clinical importance indicating the necessity to continue ^177^Lu-PSMA-617 therapy even if patients have failed to respond to the first cycle, and allows also the conclusion that higher therapy doses should be used, at least along with the first cycle. Yordanova et al. [16] recently reported a clinical benefit in 30 ^177^Lu-PSMA-617 re-treated patients with a median OS of 25 months versus 9 months in patients who received only one ^177^Lu-PSMA-617 treatment period, each period consisting of several treatment cycles. Similar beneficial results were reported for ^177^Lu-PSMA-I&T re-treatment by Grubmüller et al. [17] and Gafita et al. [18]. Basically, these retrospective analyses raise the question whether higher activities than 6 GBq of ^177^Lu-PSMA-617 would fit more appropriate as Grade 4 toxicity is rare, even in rechallenged patients, with reasonable efficacy.

Despite of the increasing world-wide use of PSMA Ligand Radionuclide Therapy (PLRT), published survival data on ^177^Lu-PSMA-617-treated mCRPC patients are still rare. Basically, our data on PFS and OS in consecutive patients are less pronounced compared with those of some other centers reporting retrospective analyses which might be partly due to heavier pre-treatment of our patients. In their initial report in 56 patients, Baum et al. [19] reported a PFS of 13.7 months and a OS of 15.5 months using ^177^Lu-PSMA-I&T. In a more recent study by Yadav et al. [20] in 31 patients, the median PFS was 12 months and the median OS was 16 months. While these numbers on OS compare well with docetaxel-based results [21], our recent meta-analysis [22] in mCRPC patients was more favorable for PSMA-targeted radioligand therapy (PRLT) with approximately 43% PSA response versus 22% in third-line chemotherapy-treated patients. In addition, therapy with ^177^Lu-PSMA-617 does not only work better than third-line chemotherapy but also works after third-line chemotherapy with prolonged survival [23].

The results of this study in 32 consecutive patients show that patients receiving at least 2 therapy cycles of ^177^Lu-PSMA-617 (i.e., 12 GBq) and responding to therapy—and these are roughly 2/3 of patients—live 6 months longer than “non-responders.” Though this result has to be taken with caution due to the rather small number of patients reported herein, it clearly underlines the current hype for this type of therapy for mCRPC patients. A significant difference in median OS between responders and non-responders for a change in PSA was also reported by Ahmadzadehfar et al. [24] in 52 patients showing a survival benefit of 9 months.

In the presently only available prospective Phase II single-center study in 30 mCRPC patients by Hofman et al. [25], the estimated PFS was 7 months and the median OS was 13.5 months which compares favorably with our single-center cohort of 32 patients who were consecutively assigned for treatment and who were followed until death. Despite of the similar study design between both our centers, at this advanced stage of PC disease of heavy pre-treatments, the results of the presently recruiting prospective VISION phase 3 trial [26] will be very important as this trial is designed randomized against best supportive care. As it will take years for long-term survival results of the VISION trial, the WARMTH (World Association of Radiopharmaceutical and Molecular Therapy) initiative of gathering all available retrospective data on ^177^Lu-PSMA-617 into a database [27] will hopefully bring more light earlier into important issues of PRLT. Issues consist in several open questions such as response prediction, and thus patient selection [28, 29].

The assessment of treatment response is challenging while being critical in the oncological practice. RECIST performs well in assessment of tumor shrinkage as a criteria for response. PERCIST based on ^68^Ga-PSMA-11 PET/CT may perform better than RECIST in a patient with PSA progression. ^68^Ga-PSMA-11 PET/CT plays an important role in patient selection and probably in predicting treatment response to ^177^Lu-PSMA-617. In their recent report on the value of ^68^Ga-PSMA-11 PET/CT, Emmett el al. [30] found that an increased SUV_max_ value of the tumor lesions goes along with the prediction of treatment response in terms of PSA reduction. While by large variation our SUV_max_ values decreased under therapy in PSA-responding patients, we could not find a significant correlation for neither PFS nor OS. On the other hand, a reduced tracer uptake may not necessarily translate into a longer survival time. In fact, the results of our evaluation indicate a good agreement of the visual interpretation of ^68^Ga-PSMA-11 PET/CT and 24 h WB scans and implicate the value of post-therapy imaging. Moreover, serial PSA measurements together with 24 h WB scintigraphy seem to be sufficiently accurate for the follow-up of ^177^Lu-PSMA-617-treated patients. Of course, more lesions can be seen on ^68^Ga-PSMA-11 PET/CT compared to 24 h WB scintigraphy. However, very often it is rather difficult to count numerous lesions on PET/CT as well as on 24 h WB scans in the individual patient. It is also time-consuming, expensive, and sometimes difficult to perform repeated ^68^Ga-PSMA-11 PET/CT in heavily bone-metastasized patients, who often cannot endure the imaging acquisition time due to bone pain. In contrast, the performance of 24 h WB scintigraphy after therapy is simple and cost-effective and seems accurate enough to allow a conclusion on response to therapy—especially together with PSA measurements. RECIST [2] is based on cross-sectional abdominopelvic imaging together with bone scintigraphy and PSA serum levels. While ^68^Ga-PSMA-11 PET/CT has a higher sensitivity especially in patients with lower PSA values than any other imaging modality [3], it may give also controversial in patients with dedifferentiated tumors. In fact, Heinzel, et al. [31] recently reported a moderate sensitivity of 85% and a specificity of about 60% only for ^68^Ga-PSMA-11 PET/CT for monitoring ^177^Lu-PSMA-617 therapy.

Calculation of the absorbed tumor dose may be an important predictor for clinical response. In our mCRPC patients, the absorbed dose of ^177^Lu-PSMA-617 of tumor lesions was significantly higher than that of normal organs, especially kidneys and bone marrow. Despite the fact that a mCRPC patient may show variable uptake of ^177^Lu-PSMA-617 in the multiple tumor lesions, dosimetry occurs clinically relevant for healthy organs. A recent retrospective analysis in a total of 167 patients indicated that taxane chemotherapy pre-treated patients will also benefit from ^177^Lu-PRLT treatment with rare Grade 3 or 4 toxicity [32]. In fact, in all retrospective reports on ^177^Lu-PSMA-617 [20, 24, 33–38] or ^177^Lu-PSMA-I&T [17–19, 39], hematotoxicity and nephrotoxicity were rare, and the treatment safe even in patients with a single kidney [40]. In fact, with the kidneys presenting the critical organ, a cumulative activity of 30 GBq of ^177^Lu-PSMA-617 appears to be safe and justifiable. A recent study by Okamoto et al. [41] using ^177^Lu-PSMA-I&T demonstrated gradually decreasing tumor dose estimates from one cycle to the other and correlation between pre-therapeutic SUV values and absorbed tumor dose estimates. In our study, only a weak correlation was found for SUV_max_ values of LN metastases with absorbed dose estimates but not for skeletal and visceral metastases. Whereas dose estimates were similarly high with values of around 3 Gy/GBq, an explanation of this divergent finding between the Munich group and out center could lie in the use of different PSMA ligands. Also Zang et al. [42] reported only a moderate correlation of SUV_max_ values in a limited number of patients, both for ^177^Lu-PSMA-617 and ^177^Lu-EB-PSMA-617. Violet et al. [43] estimated a significant correlation between SUV_mean_ of the tumor lesions and the mean absorbed dose and reduced salivary and kidney doses in patients with higher tumor burden. Probably patients with pre-dominant LN-mCRPC may benefit from ^177^Lu-PSMA-617 therapy the most [44] as especially skeletal metastases may show MX responses more often. In this study, all patients with a MX response also showed PSA progression over time. While one would expect a correlation of SUV_max_, TU/BG ratios, and/or tumor dose estimates at baseline, there are factors which may explain the divergent finding of this study cohort in a limited number of 32 patients. First, the radiotracers used for imaging (PSMA-11) and therapy (PSMA-617) are not exactly the same, despite of being similar. Second, especially smaller lesions are problematic for absorbed dose calculation due to the partial volume effect. This is why in Fig. 3, we have tried to exclude tumor lesions with a volume < 7 ml; however, we could not find a correlation for our cohort. Furthermore, the variety of different pre-treatments, ongoing bone-targeted therapy, or heterogeneity of tumor lesions may also count for divergent findings which might be better ruled out in a future study involving a higher number of patients.

In 8/32 (25%) patients of this study, the only mentionable side effect was transient or permanent xerostomia (Table 4), despite of a somewhat higher incidence rate than reported in the German retrospective multicenter study [45]. In roughly 2/3 of patients, not only in the WB studies but also in the PET/CT studies, a decreased uptake was found along with treatment. The dosimetric calculations for salivary glands (Table 5) show a trend for correlation of SUV_max_ and estimated absorbed dose values for salivary glands, parotid as well as submandibular glands, both in line with volume reduction of the glands after up to 4 therapy cycles. This observation indirectly outlines the concept of irradiation effects on tumor volume resulting in tumor shrinkage after radioisotope irradiation. In fact, several interventions have been discussed for salivary gland protection including the use of cool bags [46], botox, and steroid injection [47]. A similar high rate of appearance of xerostomia was mentionably reported in the prospective study with ^177^Lu-PSMA-617 and was attributed to specific questioning of this potential toxicity within the trial setting [25]. Grade 2 xerostomia was also reported for 17% of the patients in the evaluation of Kalmthout et al. [48] whereas in the German multicenter study it occurred in 8% only [45]. With newer developments of PRLT, especially ^225^Ac-PSMA-617, the radiation-induced effects on salivary gland function must be taken seriously and seem to be much higher [49, 50]. Furthermore, a recent report by Violet et al. [43] showed reduced salivary doses in patients with higher tumor burden, increased body mass, and body surface area, referred to as “tumor-sink” effect and providing a rational personalized treatment dosing. Personalization of treatment seems to be relevant also for hematotoxicity in patients with diffuse red marrow infiltration and extensive chemotherapeutic pre-treatments [38, 51].

Finally, the application of PRLT as opposed to the guideline-recognized ^223^Ra-therapy in mCRPC patients [52] should gain better overall results, probably with a treatment response longer than 3.6 months, highlighting the advantage of a tumor-targeted ligand rather than exclusively bone-seeking agent.

## Limitations of the study

Whereas our results from a single center in prospective consecutive manner direct for future study planning, the results have to be confirmed in future-controlled studies. Our center evaluation underlines that PSMA-directed therapy has the potential to change the clinical management of mCRPC patients. Several prospective PSMA-targeting studies are in progress of which the VISION study [25] will provide data on OS as the primary endpoint versus best standard of care. An important question if patients with a longer PFS also have a longer OS cannot be answered from the current study due to the limited patient number studied. Furthermore, in this study, neither initial PSA values nor initial TU/BG ratios and SUV_max_ values correlated with PFS and OS. This again can be due to the rather low number of patients included and significantly outlines the importance of prospective study results.

## Electronic supplementary material

ESM 1 (PDF 798 KB)

ESM 2 (PDF 798 KB)

ESM 3 (PDF 798 KB)

ESM 4 (PDF 798 KB)

ESM 5 (PDF 798 KB)

ESM 6 (DOCX 23 kb)
