# Supplementary material for: The 68Ga/177Lu-theragnostic concept in PSMA-targeting of metastatic castration–resistant prostate cancer: impact of post-therapeutic whole-body scintigraphy in the follow-up
Source: Eur J Nucl Med Mol Imaging. 2019 Nov 27;47(3):695–712. doi: 10.1007/s00259-019-04583-2 (PMC7005064; doi:10.1007/s00259-019-04583-2)
Supplement: Supplementary file 1 — (PDF 798 KB) [file 259_2019_4583_MOESM1_ESM.pdf]

The ^{68}Ga / ^{177}Lu-Theragnostic Concept in PSMA-Targeting of Metastatic Castration Resistant Prostate Cancer: Impact of Post-therapeutic Whole Body Scintigraphy in the Follow-Up

Maffey-Steffan J.[1]*, Scarpa L[1]*, Svirydenka A[1]*, Nilica B[1], Mair C[1], Buxbaum S[1], Bektic J[2], von Guggenberg E[1], Uprimny C[1], Horninger W[2], Virgolini I[1]

Departments of [1]Nuclear Medicine and [2]Urology, Medical University of Innsbruck, 6020 Innsbruck, Austria.
*the three authors contributed equally to the manuscript

Corresponding Author: Irene J. Virgolini, M.D., Director, Department of Nuclear Medicine, Medical University Innsbruck, Anichstraße 35, A-6020 Innsbruck, Tel: +43 (0)50 504 22650 or ext. 80935, FAX: +43 (0)50 504 22659, E-Mail: irene.virgolini@i-med.ac.at, http://nuklearmedizin-innsbruck.com

Supplement 1: Demographic Data of Metastatic Resistant Prostate Cancer Patients

Patient	Age	Operations	Radiotherapy	Chemotherapy	Hormonal Therapy	Supportive Therapy
1	75	prostatectomy, vesiculectomy	EBRT	/	bicalutamide, degralix	zoledronic acid◆
2	76	prostatectomy	/	/	leuprorelin	/
3	82	prostatectomy	proton therapy	/	leuprorelin, enzalutamide	denosumab◆
4	61	/	EBRT	docetaxel	bicalutamide, degralix, dutasteride, enzalutamide	24.4 ^{223}Ra (MBq)
5	68	/	/	/	bicalutamide, leuprorelin	/
6	63	prostatectomy	EBRT	/	abiraterone, leuprorelin	18.1 ^{223}Ra (MBq), denosumab◆
7	74	prostatectomy, decompression L4/L5	/	/	gosereline, enzatulamide	zoledronic acid◆
8	59	prostatectomy	/	docetaxel	bicalutamide, tamoxifen, abiraterone, enzalutamide	zoledronic acid, denosumab◆
9	56	/	/	docetaxel	degarelix, pamoreline, leuprorelin	26.3 ^{223}Ra (MBq), denosumab
10	67	prostatectomy, vesiculectomy	/	docetaxel	triptoreline, leuprorelin	denosumab
11	89	prostatectomy, vesiculectomy	EBRT	docetaxel	enzalutamide, abiraterone	denosumab
12	68	/	/	docetaxel	enzalutamide, abiraterone, leuprorelin, bicalutamide	denosumab
13	59	/	/	docetaxel	bicalutamide, leuprorelin, triptorelin	denosumab
14	91	/	EBRT	docetaxel	enzalutamide, abiraterone, bicalutamide, gosereline, degralix	denosumab◆
15	81	prostatectomy	EBRT	docetaxel	enzalutamide, abiraterone, bicalutamide, gosereline, degralix	denosumab
16	60	prostatectomy, vesiculectomy	/	docetaxel	abiraterone	/
17	80	/	EBRT	/	enzalutamide, abiraterone	/
18	73	prostatectomy, vesiculectomy	/	docetaxel	abiraterone, degralix, triptoreline	24.76 ^{223}Ra (MBq)
19	75	/	/	docetaxel	enzalutamide, degralix, leuprorelin	denosumab
20	60	prostatectomy, vesiculectomy	EBRT	/	abiraterone, bicalutamide, gosereline	alendronic acid, denosumab◆
21	82	/	EBRT	docetaxel	enzalutamide, bicalutamide, leuprorelin, degralix	/
22	75	/	EBRT	/	/	/
23	71	prostatectomy, vesiculectomy	EBRT	docetaxel	enzalutamide, bicalutamide, leuprorelin	cannabis, denosumab
24	69	/	/	docetaxel	enzalutamide, leuprorelin	denosumab
25	73	palliative transurethral resection	EBRT, proton therapy	regional chemotherapy	/	/
26	84	prostatectomy, vesiculectomy	salvage radiation	docetaxel	enzalutamide	22.91 ^{223}Ra (MBq)
27	61	prostatectomy	EBRT	docetaxel	bicalutamide, enzalutamide, abirateron, cyproteronacetate	/
28	83	prostatectomy, penectomy	EBRT	docetaxel	bicalutamide, leuprorelin, abirateronacetate	/
29	73	/	/	cabazitaxel, docetaxel	degarelix, trenantone, bicalutamide, tamoxifen	/
30	50	/	/	cisplatin/etoposide	bicalutamide, leuprorelin, leuprorelinacetate	denosumab
31	67	prostatectomy	/	docetaxel, cabazitaxel	bicalutamide, leuprorelin, enzalutamide, arbiterone	/
32	79	prostatectomy	/	/	bicalutamide, enzalutamide, leuprorelin	denosumab◆

EBRT: External beam radiation therapy

◆ Therapy ongoing
