# Supplementary material for: The 68Ga/177Lu-theragnostic concept in PSMA-targeting of metastatic castration–resistant prostate cancer: impact of post-therapeutic whole-body scintigraphy in the follow-up
Source: Eur J Nucl Med Mol Imaging. 2019 Nov 27;47(3):695–712. doi: 10.1007/s00259-019-04583-2 (PMC7005064; doi:10.1007/s00259-019-04583-2)
Supplement: Supplementary file 2 — (PDF 798 KB) [file 259_2019_4583_MOESM2_ESM.pdf]

Supplement 2 – Dosimetry Calculations:

For each OAR and tumor the background was subtracted from the total counts and subsequently the mean counts per pixel were calculated. In case of tumors and abdominal OAR the background counts, which were established near the femur, were used for background correction. However, for salivary glands the ROI at the forehead was subtracted to establish an appropriate background correction. Voluminous organs, i.e. the liver, were background corrected by using only half of the background counts to guarantee an adequate proportionality. Subsequently a correction for overlap of tumors or organs was applied to the data if necessary. By means of the logged count rate during the whole-body-scans for each post-injection measurement and for the recorded corresponding time points, the initial activity could be calculated backward, making the assumptions of a linear behavior of the decay and no loss of activity before the first scan at 30 minutes post-injection. Thus the cumulated fraction of the initial activity in the patient was plotted as a function of time. The resulting time-activity-curves (TACs) were fitted by means of a three exponential decay function. In addition, the TAC of the remaining whole body (WB-remainder) after the subtraction of all OAR and tumors was approached by means of a two-exponential function. Integrating the resulting TACs lead to the demanded residence times for each organ and tumor as well as the remainder.

The residence times of liver, kidneys, bladder, spleen and remainder were assumed as source organs in OLINDA/EXM [13] to calculate the corresponding doses per injected activity. The standardized phantom organ masses determined by OLINDA were modified by a calculated correction factor, established by means of the body mass index of the patient. The kidneys received special consideration when it came to mass evaluation. Therefore the kidney volumes were determined using the planning software PINNACLE and a pretherapeutic CT-scan which should guarantee better individual accuracy for this organ than the standardized setting of OLINDA. To calculate the tumor dose and the dose of the salivary glands a spherical model was used for individual volumes derived from previous PET/CT images acquired before the first treatment cycle and SPECT/CT images acquired 24 hours post injection.

The reported tumor absorbed doses for skeletal, visceral and lymph node metastases represent the average of all metastases of each type of metastases for each patient. Organ doses were calculated under the assumption of a constant tracer uptake of tumors and OAR over all treatment cycles. In contrast to the previous study by Kratochwil et al. [34] background correction was performed also for tumors, to avoid an overestimate of tumor absorbed doses. To evaluate red marrow dose the calculated remainder dose from OLINDA was used and modified by a correction factor, which was determined by means of blood dosimetry. The latter gave information about the impact of the blood pool activity on the red marrow dose and provided an empirical value of 20%. Thus the calculated red bone marrow dose using OLINDA was increased by a factor of 20% for each patient.
