# Supplementary material for: The 68Ga/177Lu-theragnostic concept in PSMA-targeting of metastatic castration–resistant prostate cancer: impact of post-therapeutic whole-body scintigraphy in the follow-up
Source: Eur J Nucl Med Mol Imaging. 2019 Nov 27;47(3):695–712. doi: 10.1007/s00259-019-04583-2 (PMC7005064; doi:10.1007/s00259-019-04583-2)
Supplement: Supplementary file 3 — (PDF 798 KB) [file 259_2019_4583_MOESM3_ESM.pdf]

Supplement 3: ECOG Evaluation						
	Cycles					
Patient	1.	2.	3.	4.	5.	6.
1	0	0	0			
2	0	0	0	0		
3	0	0	0	1		
4	0	0	0			
5	0	0	0	0		
6	0	1	0	0		
7	0	0	1			
8	0	0				
9	0	0	0			
10	0	0	0	0	0	0
11	0	0	1	1	1	1
12	0	0				
13	0	0	0	0		
14	1	1	1	1		
15	1	2	2			
16	1	1	1	1		
17	0	0	0	0		
18	0	1	1			
19	1	1	1	1		
20	0	0	0	0	0	0
21	1	1				
22	0	0	0	0		
23	0	2	2	2		
24	0	0	1	1		
25	0	0	0	0		
26	0	0	0	0		
27	0	0	0			
28	0	0	0	0	0	
29	1	1	1			
30	1	1	1	1		
31	0	0	0	0		
32	0	0	0	0		
1. = Baseline Performance Status obtained before administration of the 1st therapy cycle						
2. = Performance Status obtained 6-10 weeks after the 1st cycle						
3. = Performance Status obtained 6-10 weeksafter the 2nd cycle						
4. = Performance Status obtained 6-10 weeks after the 3rd cycle						
5. = Performance Status obtained 6-10 weeksafter the 4th cycle						
6. = Performance Status obtained 6-10 weeksafter the 5th cycle						
