# Supplementary material for: The 68Ga/177Lu-theragnostic concept in PSMA-targeting of metastatic castration–resistant prostate cancer: impact of post-therapeutic whole-body scintigraphy in the follow-up
Source: Eur J Nucl Med Mol Imaging. 2019 Nov 27;47(3):695–712. doi: 10.1007/s00259-019-04583-2 (PMC7005064; doi:10.1007/s00259-019-04583-2)

Supplement 4: Correlation of SUV_{max} and TU/BG ratios and PSA, and TU/BG ratios and PSA-values.

The Pearson's correlation coefficient was applied to compare the different response assessment criteria, but no correlation could be found between neither SUV_{max} and TU/BG, SUV_{max} and PSA, nor Tu/BG and PSA-values.

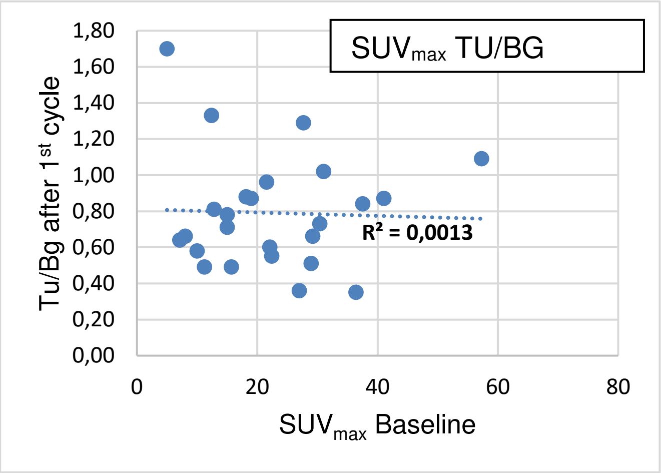

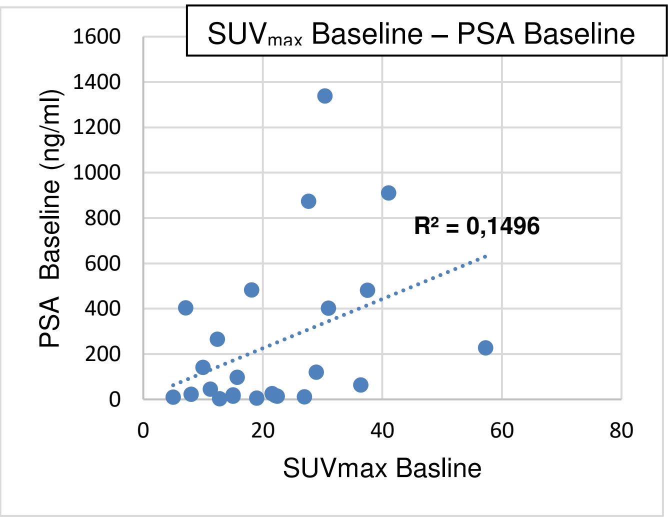

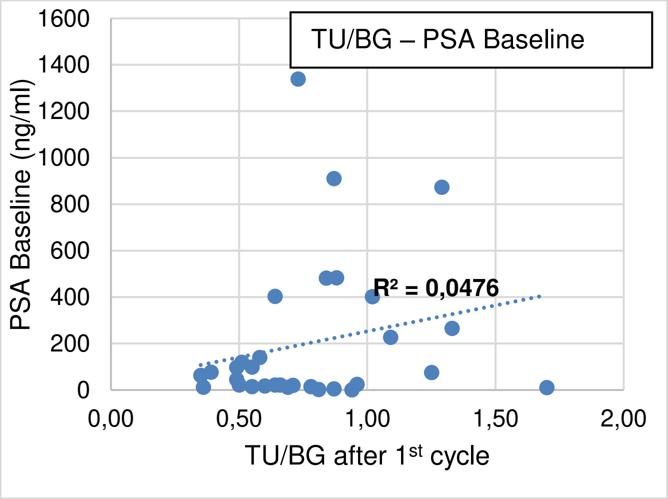

Supplement: Supplementary file 4 — (PDF 798 KB) [file 259_2019_4583_MOESM4_ESM.pdf]
