# Supplementary material for: The 68Ga/177Lu-theragnostic concept in PSMA-targeting of metastatic castration–resistant prostate cancer: impact of post-therapeutic whole-body scintigraphy in the follow-up
Source: Eur J Nucl Med Mol Imaging. 2019 Nov 27;47(3):695–712. doi: 10.1007/s00259-019-04583-2 (PMC7005064; doi:10.1007/s00259-019-04583-2)

Supplement 5: The Cox proportional hazards regression analysis was performed in order to evaluate if the covariates „SUV_{max} at baseline“, „PSA values at baseline“ as well as „absorbed dose of skeletal metastases“ have a significant influence on OS. For our cohort, the Chi-squared test of the log-likelihood ratio, which tries to evaluate if the model in use differs from a simpler model without influence of the covariates, led to a probability larger than 0.05. This indicates that the covariates deliver no significant information. Furthermore, the p-values of the regression coefficients also indicated that none of the variables has an impact on OS.

Test of the null hypothesis H0: beta=0:

Statistic	DF	Chi-Square	Pr > Chi²
-2 Log(Likelihood)	3	2,81381802	0,421
Score	3	3,309806612	0,346
Wald	3	3,096493053	0,377

Regression coefficients:

Variable	Value	Standard error	Wald-Chi-Square	Pr > Chi²	Hazard ratio	Hazard ratio lower boundary (95%)	Hazard ratio upper boundary (95%)
absorbed dose skel. Met. [Gy/GBq]	0,077	0,093	0,685	0,408	1,080	0,900	1,297
PSA baseline	0,000	0,001	0,263	0,608	1,000	0,999	1,002
SUVmax baseline	0,019	0,022	0,724	0,395	1,019	0,976	1,065

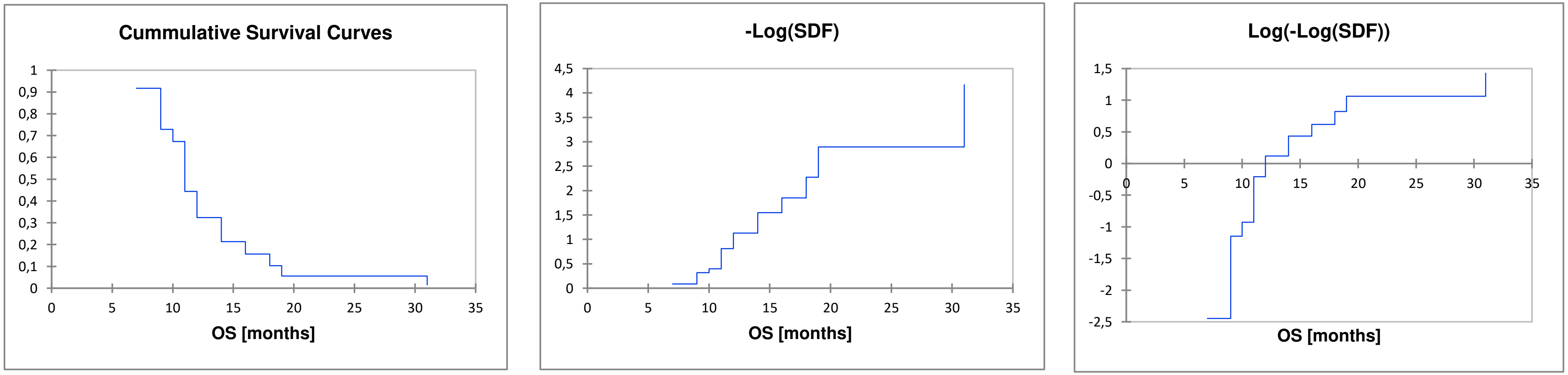

Supplement: Supplementary file 5 — (PDF 798 KB) [file 259_2019_4583_MOESM5_ESM.pdf]
