# Supplementary material for: The 68Ga/177Lu-theragnostic concept in PSMA-targeting of metastatic castration–resistant prostate cancer: impact of post-therapeutic whole-body scintigraphy in the follow-up
Source: Eur J Nucl Med Mol Imaging. 2019 Nov 27;47(3):695–712. doi: 10.1007/s00259-019-04583-2 (PMC7005064; doi:10.1007/s00259-019-04583-2)
Supplement: Supplementary file 6 — (DOCX 23 kb) [file 259_2019_4583_MOESM6_ESM.docx]

| **Test of the null hypothesis H0: beta=0:** | |  |  |
| --- | --- | --- | --- |
|  |  |  |  |
| Statistic | DF | Chi-Square | Pr > Chi² |
| -2 Log(Likelihood) | 3 | 2,81381802 | 0,421 |
| Score | 3 | 3,309806612 | 0,346 |
| Wald | 3 | 3,096493053 | 0,377 |

| **Regression coefficients:** |  |  |  |  |  |  |  |  |
| --- | --- | --- | --- | --- | --- | --- | --- | --- |
|  |  |  |  |  |  |  |  |  |
| Variable | Value | Standard error | Wald-Chi-Square | Pr > Chi² | Hazard ratio | Hazard ratio lower boundary (95%) | Hazard ratio upper boundary (95%) |  |
| absorbed dose skel. Met. [Gy/GBq] | 0,077 | 0,093 | 0,685 | 0,408 | 1,080 | 0,900 | 1,297 |  |
| PSA baseline | 0,000 | 0,001 | 0,263 | 0,608 | 1,000 | 0,999 | 1,002 |  |
| SUVmax baseline | 0,019 | 0,022 | 0,724 | 0,395 | 1,019 | 0,976 | 1,065 |  |

**Supplement 6**: Cox Proportional Hazards Regression Analysis
